# CRISPR-Cas12a-Based Detection of SARS-CoV-2 Harboring the E484K Mutation

**DOI:** 10.1021/acssynbio.1c00323

**Published:** 2021-11-16

**Authors:** María-Carmen Marqués, Raúl Ruiz, Roser Montagud-Martínez, Rosa Márquez-Costa, Sandra Albert, Pilar Domingo-Calap, Guillermo Rodrigo

**Affiliations:** † Institute for Integrative Systems Biology (I2SysBio), CSIC − University of Valencia, 46980 Paterna, Spain

**Keywords:** CRISPR diagnostics, epidemiological surveillance, virus evolution

## Abstract

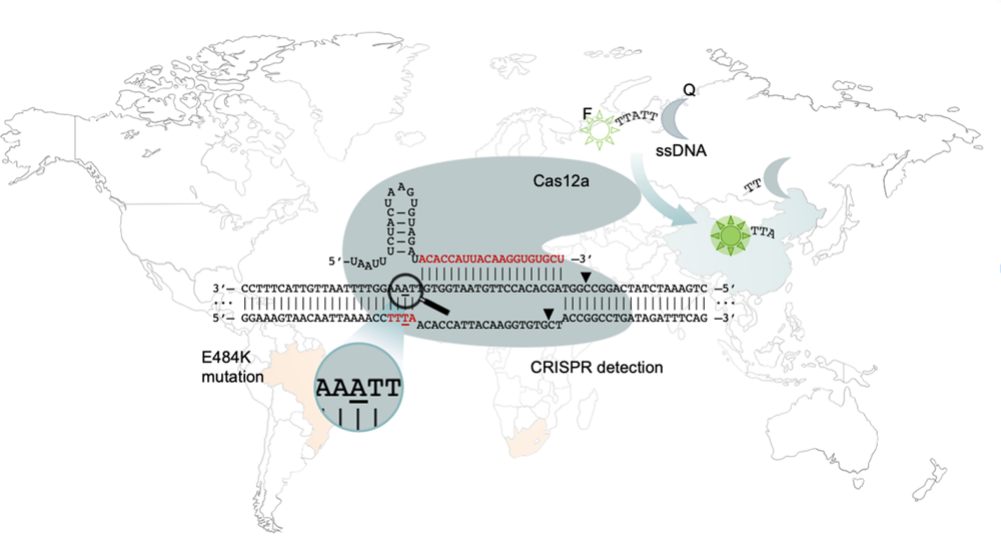

The novel respiratory virus SARS-CoV-2 is rapidly evolving across the world with the potential of increasing its transmission and the induced disease. Here, we applied the CRISPR-Cas12a system to detect, without the need of sequencing, SARS-CoV-2 genomes harboring the E484K mutation, first identified in the Beta variant and catalogued as an escape mutation. The E484K mutation creates a canonical protospacer adjacent motif for Cas12a recognition in the resulting DNA amplicon, which was exploited to obtain a differential readout. We analyzed a series of fecal samples from hospitalized patients in Valencia (Spain), finding one infection with SARS-CoV-2 harboring the E484K mutation, which was then confirmed by sequencing. Overall, these results suggest that CRISPR diagnostics can be a useful tool in epidemiology to monitor the spread of escape mutations.

Severe acute respiratory syndrome coronavirus 2 (SARS-CoV-2) has caused the coronavirus disease 2019 (COVID-19) pandemic, which has highlighted the challenges in diagnostics of viral infections, especially when a fast, massive, and reliable intervention is required to reduce the transmission.^1^ An additional problem is the emergence with time of novel SARS-CoV-2 variants that harbor specific mutations with the potential of increasing transmission of the virus and the induced disease, thereby posing concerns about the mitigation of the pandemic.^2^ In this regard, sequencing approaches are being applied worldwide to monitor the spread of the virus.^3^ However, this is a time-consuming and expensive approach that needs to be complemented with shallower and simpler techniques to maximize population testing.

In recent years, clustered regularly interspaced short palindromic repeats (CRISPR) systems have been repurposed for diagnostic applications thanks to the nonspecific collateral catalytic activity of the Cas proteins upon the specific RNA-guided targeting of the nucleic acid of interest.^4^ Owing to the exquisite sequence specificity of these systems, they can even be exploited to discriminate mutants.^5^ One mutation in SARS-CoV-2 that has gained attention is E484K, a substitution of a glutamic acid by a lysine in the receptor-binding domain of the spike protein (at the position 484 of the protein). This mutation was first identified in the Beta variant of concern (emerged in South Africa),^6^ but later it was also identified in the Gamma variant (emerged in Brazil) and in an evolved version of the Alpha variant (emerged in UK), which represent independent lineages (Figure 1A). The E484K mutation is an innovation that seems to be associated with an increased transmissibility and also with an escape from neutralizing antibodies.^7^

**Figure 1:**
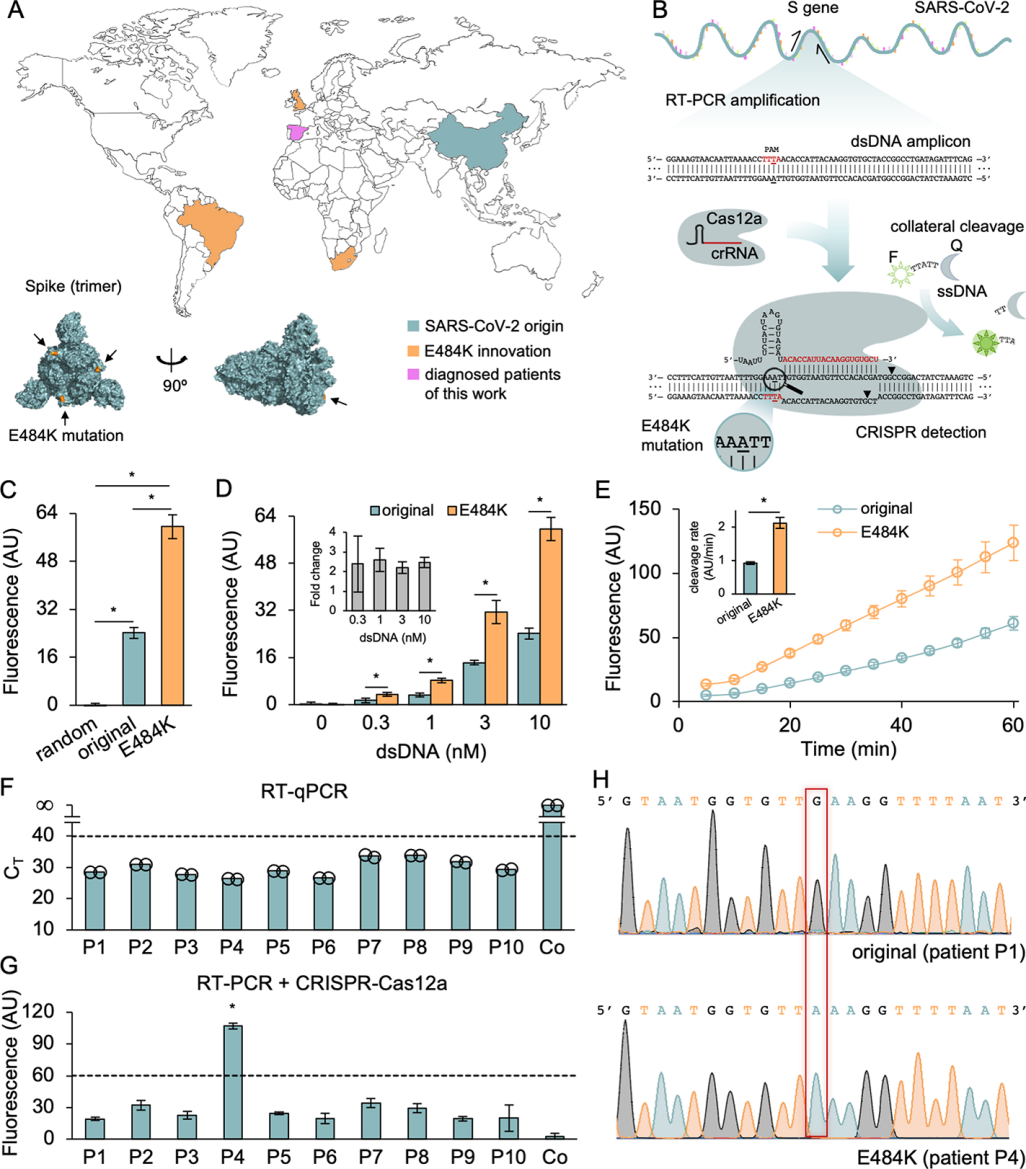
CRISPR-Cas12a-based detection of SARS-CoV-2 harboring the E484K mutation. (A) World map showing the origin of SARS-CoV-2 (in China) and the appearance of the E484K mutation in the variants of concern Beta (in South Africa), Gamma (in Brazil), and evolved Alpha (in UK). The patient samples analyzed in this work are from Valencia (Spain). On the bottom, structural model of the spike protein (mutation E484K colored in orange and pointed by an arrow). (B) Schematics of the reaction of amplification by RT-PCR and detection by CRISPR-Cas12a. A dsDNA amplicon from the SARS-CoV-2 S gene was generated with appropriately designed primers. The E484K mutation creates a PAM sequence for Cas12a recognition in the resulting dsDNA amplicon. (C) Fluorescence-based characterization of the detection with synthetic dsDNA molecules (at 30 min). (D) Fluorescence-based characterization of the detection with synthetic dsDNA molecules at different concentrations (at 30 min). In the inset, fold change in fluorescence upon detection of the E484K mutation. (E) Time-course characterization of the detection (synthetic dsDNA at 10 nM). In the inset, collateral cleavage rate, as the slope of the linear regression between fluorescence and time (from 10 to 40 min). (F) Detection of SARS-CoV-2 (N gene) in patient samples by RT-qPCR with the CDC primers (*n* = 2). A *C*_T_ of infinity means no amplification. Co is a control sample without virus. (G) Detection of SARS-CoV-2 (S gene) by RT-PCR followed by CRISPR-Cas12a. Error bars correspond to standard deviations in all cases (*n* = 3). *Statistical significance (Welch’s *t*-test, two-tailed *P* < 0.05). (H) Sequencing chromatograms of S gene from two different patient samples. On the top, virus with the original residue in spike (patient P1). On the bottom, virus harboring the E484K mutation (patient P4). The substitution of guanine by adenine is framed in red.

In this work, we applied the CRISPR-Cas12a system, in combination with reverse transcription polymerase chain reaction (RT-PCR), to identify infections caused by variants that harbor the E484K mutation without the need of sequencing. Notably, the CRISPR-Cas12a system was already applied to detect SARS-CoV-2 in clinical samples.^8^ In the current context in which several variants of concern are emerging and causing outbreaks, enlarging its applicability to the epidemiological surveillance of key mutations seems relevant.

## Results and Discussion

At the nucleotide level, the E484K mutation is implemented as a substitution of a guanine by an adenine. As a result, three consecutive adenines appear in the SARS-CoV-2 genome in that location of the S gene (coding for the spike protein). Therefore, if a viral genome amplification process is performed, from RNA to double stranded DNA (dsDNA), a canonical protospacer adjacent motif (PAM) sequence for Cas12a recognition is generated in the resulting amplicon if the virus harbors the E484K mutation (i.e., TTCA originally and TTTA upon mutation, in the antisense strand). We exploited this fact to develop a CRISPR-Cas12a-based system to discriminate viral genomes with this mutation. We designed a CRISPR RNA (crRNA) to target the region that immediately follows this potential PAM sequence in the sense strand of the resulting amplicon (Figure 1B). A small, fluorogenic single stranded DNA (ssDNA) molecule was used as a reporter.

We characterized the activity of the designed crRNA using different synthetic dsDNA molecules as targets. Interestingly, this crRNA allowed discriminating sequences with the E484K mutation, as the fluorescence readout significantly increased as a result of the presence of the canonical PAM sequence (Figure 1C). Yet, this crRNA also allowed detecting the original viral sequence with substantial efficiency with respect to a random sequence. Arguably, Cas12a can recognize to some extent degenerated PAM sequences, especially when pyrimidines are exchanged at one position, as it is the case of TTCA.^9^ Consequently, the designed crRNA has two potential uses, one to detect the presence of SARS-CoV-2 in the sample and another to inform about if it harbors the E484K mutation. If required, it would be possible to use a Cas12a ortholog with stringent PAM recognition ability to only produce a significant fluorescence readout in the case of the mutant virus.^9^

Moreover, we characterized the system for different concentrations of the dsDNA molecule, finding consistent performance (i.e., the fold change in fluorescence was almost maintained; Figure 1D). This suggested that the discrimination of the mutant can be achieved irrespective of the efficiency of the viral genome amplification process, which is important for robust diagnostics in point-of-care applications. A kinetic characterization also showed a greater rate of fluorescence increase with time in the case of a sequence with the E484K mutation and that the CRISPR-Cas12a-based detection can be done in just 15 min (Figure 1E). The collateral cleavage rate of the ssDNA molecule roughly duplicated as a result of the presence of the canonical PAM sequence, in agreement with the end-point results presented before and suggesting double amount of active CRISPR-Cas12a-dsDNA complex in the reaction. In addition, we were able to increase the activity of the nuclease using manganese instead of magnesium in the reaction (Figure S1).^10^

Next, we applied the CRISPR-Cas12a system to analyze patient samples. We focused on fecal samples obtained from 10 hospitalized patients due to COVID-19 in Valencia (Spain) in May–June 2021. The analysis of feces is interesting because it can reveal a prolonged persistence of the virus in the patient,^11^ atop of a noninvasive sample collection. The patients were diagnosed by quantitative RT-PCR (RT-qPCR) as positive in SARS-CoV-2 infection from nasopharyngeal swabs in the hospital. First, we confirmed the presence of SARS-CoV-2 in these fecal samples by RT-qPCR amplifying the N gene (coding for the nucleocapsid protein; Figure 1F). In parallel, CRISPR-Cas12a reactions on this conserved region in the N gene were also ran to detect the virus in these patient samples (Figure S2). Then, we ran CRISPR-Cas12a reactions upon amplification of the S gene by RT-PCR. An isothermal approach could be used as well to perform the amplification to bypass the need of precise equipment.^12^ Interestingly, we found that the fluorescence readout for patient P4 was significantly higher than for the rest of patients (Figure 1G), which indicated that patient P4 was likely infected by a SARS-CoV-2 variant with the E484K mutation.

To confirm such an indication, we sequenced all dsDNA amplicons of the S gene. In a sample from patient P4, an adenine was revealed at the corresponding position (leading to the E484K mutation), while a guanine was always found in the case of all other samples (Figure 1H), in agreement with the fluorescent results. Hence, these results demonstrated that CRISPR-Cas12a reactions are useful to disclose SARS-CoV-2 infections whose genomes harbor the E484K mutation in a rapid and inexpensive way.

Conclusively, our approach relies on the formation of a suitable PAM sequence for Cas12a recognition upon mutation. To broaden its applicability, different Cas effector proteins with distinctive PAM sequence specificities, either natural or reengineered, might be used.^13^ For example, in the case of the N501Y mutation, also present in the variants Beta, Gamma, and evolved Alpha in combination with the E484K mutation,^6^ the Cas12a RVR variant might be exploited to recognize the resulting PAM sequence TATG (in the sense strand of the dsDNA amplicon).^13^ Alternatively, the promiscuity of Cas12a in the PAM recognition can be exploited (e.g., the noncanonical PAM sequence CTTA is formed upon the N501Y mutation).^9^ Considering three additional mutations that have appeared in the S gene (Figure S3), we designed suitable crRNAs to run CRISPR-Cas12a reactions with synthetically generated dsDNA amplicons, finding possible the discrimination of the mutated sequences (Figure S4).

Previous work using CRISPR-Cas systems to detect specific point mutations in SARS-CoV-2 relied on the specificity generated by the crRNA, either with Cas12a^14^ or Cas13a.^15^ These methods were used to detect the D614G mutation, which arose in the first months of the pandemic and has become dominant worldwide as a consequence of providing higher infectivity to the virus.^16^ However, the PAM-based detection represents an original approach in which the task for sequence discrimination is displaced from the crRNA to the nuclease. Accordingly, we do not need to deal with the potential tolerance to mismatches between the crRNA and the target of these systems, thereby leading to a more straightforward design process and experimental testing. Overall, CRISPR-Cas systems seem ready to be deployed in the field to complement current diagnostic procedures and, in particular, to contribute to the epidemiological surveillance of specific escape mutations in real time during a pandemic.

## Methods

### Patient Samples

Fecal samples corresponding to 10 infected patients with SARS-CoV-2 (RT-qPCR diagnostics from nasopharyngeal swabs) were obtained from the Hospital Universitario y Politécnico La Fe de Valencia (Spain). The ethics committee of the Hospital Universitario y Politécnico La Fe approved this study (registration number 2020-301-1).

### RNA Extraction

Fecal samples were resuspended in 5 mL Dulbecco’s modified Eagle’s medium (DMEM). Samples were then centrifuged twice at 3220*g* for 10 min at 4 °C to recover viruses in the supernatants. Supernatants were filtered through a 0.45 μm pore. RNA extraction was performed with the NucleoSpin RNA virus kit (Macherey-Nagel) following the manufacturer’s instructions.

### Virus Detection by RT-qPCR

The TaqPath 1-step RT-qPCR master mix, CG (Applied) was used with the Centers for Disease Control and Prevention (CDC) N1 primers to amplify the SARS-CoV-2 N gene (kit provided by IDT). In a microplate (Applied), 2 μL of RNA sample were mixed with 500 nM of primers, 125 nM of probe, and the RT-qPCR mix for a total volume of 10 μL. The microplate was placed in a real-time PCR system (QuantStudio 3, Applied) with the following protocol: 50 °C for 15 min for RT, then 45 cycles of 95 °C for 3 s for denaturation and 60 °C for 30 s for annealing and extension. Samples with cycle threshold (*C*_T_) values lower than 40 were considered as positive for SARS-CoV-2.^17^

### Virus Amplification by RT-PCR

A nested PCR approach was followed to enhance the specificity and increase the yield. First, RT reactions were performed using 400 ng of total RNA (previously denatured at 65 °C for 5 min) with 500 nM of primary reverse primer to amplify the S gene, 1 nM dNTPs (NZYTech), 10 U/μL RevertAid (Thermo), and 1 U/μL RNase inhibitor (Thermo). This reaction was incubated at 42 °C for 60 min, followed by an inactivation step. Then, 2 μL of product were used as the template for the first PCR with 400 nM of the primary forward and reverse primers to amplify the S gene, 200 μM dNTPs, and 0.05 U/μL Phusion high-fidelity DNA polymerase (Thermo). Reactions were incubated in a thermocycler (Eppendorf) with the following protocol: 40 cycles of 98 °C for 10 s for denaturation, 66 °C for 10 s for annealing, and 72 °C for 10 s for extension. PCR products were then digested with an exonuclease to eliminate the remaining primers using the Illustra ExoProStar 1-step kit (Thermo). Then, 2 μL of the first PCR were used for the second PCR. The reaction conditions were the same as before, unless in this case the secondary forward and reverse primers were used, the annealing was done at 62 °C, and 45 cycles were applied.

### CRISPR-Cas Elements

ThecrRNA was generated by *in vitro* transcription with the TranscriptAid T7 high yield transcription kit (Thermo) from a DNA template. This was then purified using the RNA clean and concentrator column (Zymo) and quantified in a NanoDrop. Cas12a from *Lachnospiraceae bacterium* was a commercial preparation (NEB). The CRISPR-Cas12a ribonucleoprotein was formed by incubating in NEBuffer 2.1 (10 mM Tris-HCl, 50 mM NaCl, 10 mM MgCl_2_, 100 μg/mL BSA, pH 7.9; NEB) 62.5 nM of crRNA and 50 nM of Cas12a for 30 min at room temperature. A buffer containing MnSO_4_ instead of MgCl_2_ at the same concentration was also used.

### Virus Detection by CRISPR-Cas12a

In a microplate (Applied), 2 μL of amplified dsDNA were mixed with 17 μL of CRISPR-Cas12a ribonucleoprotein (previously formed) and 1 μL of ssDNA probe (chemically synthesized by IDT, at 500 nM). The ssDNA probe was labeled with fluorescein in the 5′ end and with a dark quencher in the 3′ end. The microplate was placed in a real-time PCR system (QuantStudio 3, Applied), incubating for 1 h at 37 °C and measuring green fluorescence each 5 min. Excitation was at 470 nm and emission at 520 nm. Represented fluorescence values correspond to absolute signals minus the background signal obtained in absence of dsDNA.

### Virus Sequencing

Amplified dsDNA molecules from the SARS-CoV-2 S gene were sent to Eurofins Genomics for Sanger sequencing with the primary forward primer.

## Supporting Information Available

Supporting figures and tables; Sequences of all nucleic acids used in this work provided in Table S1; Information about the RT-qPCR experiments [17] provided in Table S2 (PDF)
