# Supplementary material for: CRISPR-Cas12a-Based Detection of SARS-CoV-2 Harboring the E484K Mutation
Source: ACS Synth Biol. 2021 Nov 16;10(12):3595–9. doi: 10.1021/acssynbio.1c00323 (PMC8610009; doi:10.1021/acssynbio.1c00323)
Supplement: Supplementary file 1 — sb1c00323_si_001.pdf [file sb1c00323_si_001.pdf]

Supplementary Information

CRISPR-Cas12a-based detection of SARS-CoV-2 harboring the E484K mutation

María-Carmen Marqués[1], Raúl Ruiz[1], Roser Montagud-Martínez[1], Rosa Márquez-Costa[1], Sandra Albert[1], Pilar Domingo-Calap[1], and Guillermo Rodrigo[1,*]

[1]Institute for Integrative Systems Biology (I2SysBio), CSIC – University of Valencia, 46980 Paterna, Spain

*Correspondence: guillermo.rodrigo@csic.es

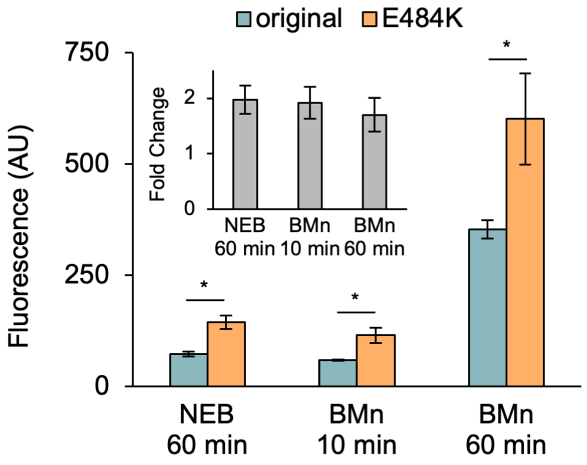

Figure S1: Effect of the metal on the CRISPR-Cas12a-based detection. Fluorescence-based characterization of the detection with synthetic dsDNA molecules. NEB means reaction buffer with Mg^{2+} and BMn buffer with Mn^{2+}. *Statistical significance (Welch's *t*-test, two-tailed $P < 0.05$). In the inset, fold change in fluorescence upon detection of the E484K mutation.

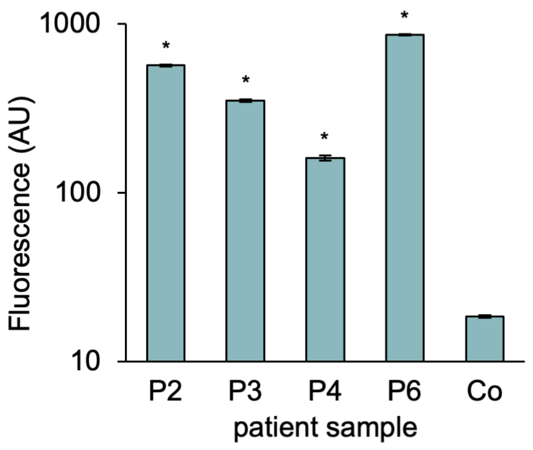

Figure S2: CRISPR-Cas12a-based detection of a conserved region in the N gene. Fluorescence-based characterization of the detection in patient samples. Amplification by RT-PCR. Error bars correspond to standard deviations in all cases (n = 3). *Statistical significance (Welch's t-test, two-tailed $P < 0.05$).

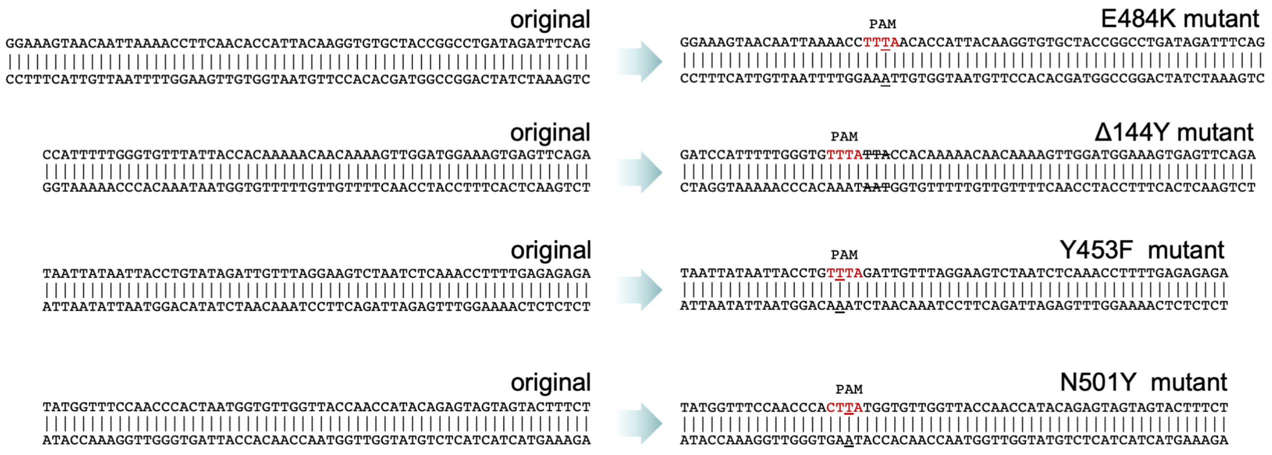

Figure S3: Sequences of regions of the SARS-CoV-2 S gene where mutations occur. The original and mutated sequences are shown. The E484K point mutation occurs in the evolved Alpha, Beta, and Gamma variants, the Δ144Y deletion in the Alpha variant, the Y453F point mutation in the B.1.1.298 variant (Denmark), and the N501Y point mutation in the Alpha, Beta, and Gamma variants. The potential PAM sequence for recognition is marked in red.

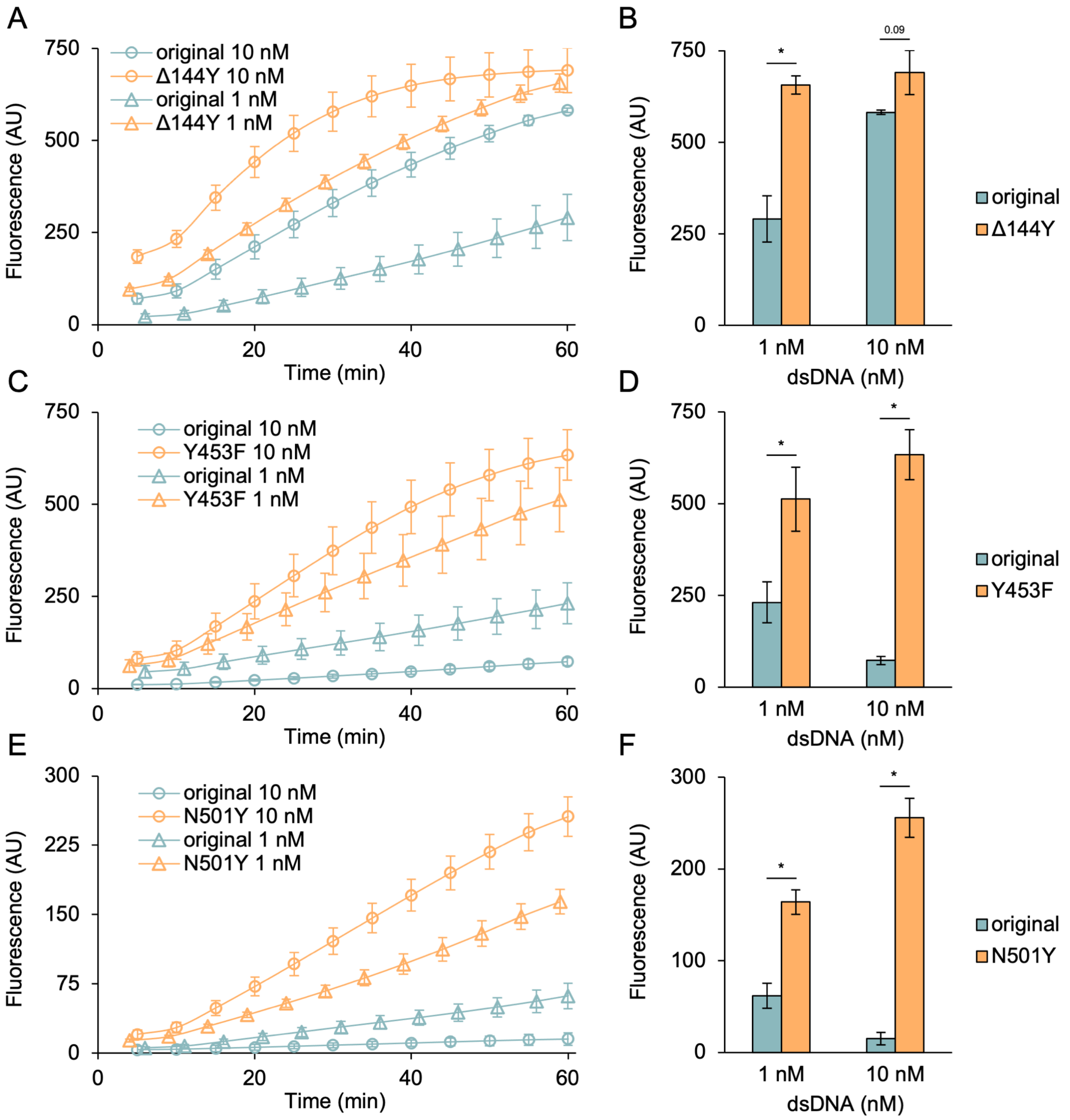

Figure S4: CRISPR-Cas12a-based detection of SARS-CoV-2 harboring other mutations. A, C, E) Time-course characterization of the detection (synthetic dsDNA at 1 or 10 nM). B, D, F) Fluorescence-based characterization of the detection with synthetic dsDNA molecules (at 1 h). Error bars correspond to standard deviations in all cases (n = 3). *Statistical significance (Welch's t-test, two-tailed $P < 0.05$).

Table S1: Sequences of all nucleic acids used in this work.

Description	Sequence (5' – 3')
forward primer to amplify N gene	GACCCCAAAATCAGCGAAAT
reverse primer to amplify N gene	TCTGGTTACTGCCAGTTGAATCTG
ssDNA probe for RT-qPCR	FAM-ACCCCGCATTACGTTTGGTGGACC-BHQ1
primary forward primer to amplify S gene	ATCGCTCCAGGGCAAACTGG
primary reverse primer to amplify S gene	ACAGTTGCTGGTGCATGTAGAAG
secondary forward primer to amplify S gene	CAGGGCAAACTGGAAAGATTG
secondary reverse primer to amplify S gene	AAAGTACTACTACTCTGTATGGTTGG
synthetic dsDNA of S gene with original residue (484E)	GAAATCTATCAGGCCGGTAGCACACCTTGTAATGGTGTTGAAG GTTTTAATTGTTACTTT
synthetic dsDNA of S gene with mutated residue (484K)	GAAATCTATCAGGCCGGTAGCACACCTTGTAATGGTGTTAAAG GTTTTAATTGTTACTTT
synthetic dsDNA of S gene with original sequence (144Y)	CCATTTTTGGGTGTTTATTACCACAAAAACAACAAAAGTTGGA TGGAAAGTGAGTTCAGA
synthetic dsDNA of S gene with deletion (Δ144)	GATCCATTTTTGGGTGTTTACCACAAAAACAACAAAAGTTGGA TGGAAAGTGAGTTCAGA
synthetic dsDNA of S gene with original residue (453Y)	TAATTATAATTACCTGTATAGATTGTTTAGGAAGTCTAATCTC AAACCTTTTGAGAGAGA
synthetic dsDNA of S gene with mutated residue (453F)	TAATTATAATTACCTGTTTAGATTGTTTAGGAAGTCTAATCTC AAACCTTTTGAGAGAGA

synthetic dsDNA of S gene with original residue (501N)	TATGGTTTCCAACCCACTAATGGTGTTGGTTACCAACCATACA GAGTAGTAGTACTTTCT
synthetic dsDNA of S gene with mutated residue (501Y)	TATGGTTTCCAACCCACTTATGGTGTTGGTTACCAACCATACA GAGTAGTAGTACTTTCT
crRNA for N1 SARS-CoV-2 detection	GGUAAUUUCUACUAAGUGUAGAUGUGGACCCUCAGAUUCAACU
crRNA for E484K mutation detection	GGUAAUUUCUACUAAGUGUAGAUACACCAUUACAAGGUGUGCU
crRNA for Δ144Y deletion detection	GGUAAUUUCUACUAAGUGUAGAUCCACAAAAACAACAAAAGUU
crRNA for Y453F mutation detection	GGUAAUUUCUACUAAGUGUAGAUGAUUGUUUAGGAAGUCUAAU
crRNA for N501Y mutation detection	GGUAAUUUCUACUAAGUGUAGAUUGGUGUUGGUUACCAACCAU
ssDNA probe for collateral cleavage by Cas12a	FAM-TTATT-IABkFQ

Table S2: Minimum information for publication of RT-qPCR experiments (MIQE; essential requirements).

**Experimental design**	
Definition of experimental and control groups	This study was performed on a cohort of 16 COVID-19 patients admitted to the Hospital Universitari i Politècnic La Fe (Valencia, Spain) between March and June 2021.
Number within each group	16 patients.
**Sample**	
Description	Stool samples were collected in 1 mL of Virocult medium and kept at 4°C until laboratory handling within 24 h.
Microdissection or macrodissection	NA
Processing procedure	Fecal samples were resuspended in DMEM to a final volume of 5 mL. Samples were then centrifuged at 3220 g for 10 min at 4°C three times to recover viruses in the supernatant. The supernatants were filtered through a 0.45 µm pore.
If frozen, how and how quickly?	-80 ºC; straight into the freezer.

If fixed, with what and how quickly?	NA
Sample storage conditions and duration (especially for FFPEb samples)	-80 ºC; 1-3 months.
**Nucleic acid extraction**	
Procedure and/or instrumentation	Lysis of viruses: Add 600 µL buffer RAV1 to 150 µL of the sample and incubate for 5 min at 70 ºC. Adjust binding conditions: Add 600 µL ethanol to lysis solution and mix. Bind viral RNA: Load 700 µL lysed sample to Nucleospin RNA Virus column and centrifuge for 1 min at 8000 g. Load the residual lysis solution onto the column and centrifuge 1 min at 8000 g. Discard flow-through. Wash and dry silica membrane: Add 500 µL buffer RAW to the column and centrifuge 1 min at 8000 g. Discard flow-through. Add 600 µL Buffer RAV3 to the column and centrifuge 1 min at 8000 g. Discard flow-through. Add 200 µL buffer RAV3 to the column and centrifuge 5 min at 11000 g. Elute viral RNA: Place the column into a 1.5 mL microcentrifuge tube, add 50 µL RNase-free H_2O (preheated to 70 ºC) and incubate for 1-2 min. Centrifuge 1 min at 11000 g and discard the column.
Name of kit and details of any modifications	Nucleospin RNA Virus Kit (Macherey-Nagel). Plant RNA Isolation Aid (Ambion) pre-treatment.
Details of DNase or RNase treatment	Buffers and Nucleospin RNA Virus columns have been tested with rRNA and MS2 phage RNA, and the absence of RNases have been investigated by RT-PCR.
Contamination assessment (DNA or RNA)	NA
Nucleic acid quantification	NA
Instrument and method	NA
RNA integrity: method/instrument	NA
RIN/RQI or C_T of 3' and 5' transcripts	NA
Inhibition testing (C_T dilutions, spike, or other)	NA
**Reverse transcription**	
Complete reaction conditions	Acording to TaqPath™ 1-Step RT-qPCR (Applied Biosystems) protocol.
Amount of RNA and reaction volume	The sample is set by volume (2 µL), not by amount of RNA. Total reaction volume of 10 µL.
Priming oligonucleotide (if using GSP) and concentration	>2019-nCoV_N1-R TCTGGTTACTGCCAGTTGAATCTG
Reverse transcriptase and concentration	Thermostable M-MLV reverse transcriptase.
Temperature and time	50 ºC, 15 min.
**qPCR target information**	
Gene symbol	N
Sequence accession number	NC_045512 REGION: 28274..29533 GeneID:43740575
Amplicon length	N1 72 bp.
In silico specificity screen (BLAST, and so on)	CDC Panel instructions.

Location of each primer by exon or intron (if applicable)	NA
What splice variants are targeted?	NA
**qPCR oligonucleotides**	
Primer sequences	CDC N1 primer set (IDT, 2019-nCoV RUO Kit)<br>>2019-nCoV_N1-F<br>GACCCCAAAATCAGCGAAAT<br>>2019-nCoV_N1-R<br>TCTGGTTACTGCCAGTTGAATCTG
Probe sequences	IDT, 2019-nCoV RUO Kit<br>>2019-nCoV_N1-P<br>ACCCCGCATTACGTTTGGTGGACC
Location and identity of any modifications	NA
**qPCR protocol**	
Complete reaction conditions	Acording to TaqPath™ 1-Step RT-qPCR protocol (Applied Biosystems).
Reaction volume and amount of cDNA/DNA	RNA: 2 µL<br>Reaction volume: 10 µL
Primer, (probe), Mg^{2+}, and dNTP concentrations	Primers: 500 nM<br>Probe: 125 nM<br>Kit includes dNTPs
Polymerase identity and concentration	Thermostable Fast DNA Polymerase
Buffer/kit identity and manufacturer	TaqPath 1-Step RT-qPCR Master Mix, CG (Applied Biosystems)
Additives (SYBR Green I, DMSO, and so forth)	NA
Complete thermocycling parameters	UNG incubation: 2 min, 25 ºC<br>Reverse transcription: 15 min, 50 ºC<br>Polymerase activation: 2 min, 95 ºC<br>Amplification (x45): 3 s, 95 ºC + 30 s, 60 ºC
Manufacturer of qPCR instrument	Applied Biosystems
**qPCR validation**	
Specificity (gel, sequence, melt, or digest)	Showed no significant combined homologies with human genome, other coronaviruses, or human microflora that would predict potential false positive RT-qPCR results.
For SYBR Green I, C_T of the NTC	NA
Calibration curves with slope and y intercept	[-3.3, -3.35] / 40.5-41.5
PCR efficiency calculated from slope	95 - 105%
R^2 of calibration curve	0.98 - 1.0
Linear dynamic range	LoD was identified by extracting and testing 10-fold serial dilutions of characterized stocks of *in vitro* transcribed RNA.
C_T variation at LOD	0.5 - 1.3
Evidence for LOD	$10^{0.5}$-10^{0} copies.
If multiplex, efficiency and LOD of each assay	NA
**Data analysis**	
qPCR analysis program (source, version)	QuantStudio Design & Analysis Software v1.5.1 (Applied Biosystems).
Method of C_T determination	Standard curve.

Outlier identification and disposition	NA
Results for NTCs	Negative.
Justification of number and choice of reference genes	NA
Description of normalization method	Passive reference (ROX).
Number and stage (reverse transcription or qPCR) of technical replicates	2 technical replicates.
Repeatability (intraassay variation)	98%.
Statistical methods for results significance	NA
Software (source, version)	NA
